# Neonatal and Infant Health Outcomes Following Maternal COVID-19 Vaccination: CDC’s COVID-19 Vaccine Pregnancy Registry

**DOI:** 10.1016/j.pedhc.2026.06.017

**Published:** 2026-07-22

**Authors:** Lauren Head Zauche, Sabrina A. Madni, David K. Shay, Christine K. Olson, Erin Stroud, Andrea J. Sharma

**Affiliations:** Division of Healthcare Quality Promotion, National Center for Emerging and Zoonotic Infectious Diseases, Centers for Disease Control and Prevention, Atlanta, GA; Division of Healthcare Quality Promotion, National Center for Emerging and Zoonotic Infectious Diseases, Centers for Disease Control and Prevention, Atlanta, GA; Division of Healthcare Quality Promotion, National Center for Emerging and Zoonotic Infectious Diseases, Centers for Disease Control and Prevention, Atlanta, GA; Division of Healthcare Quality Promotion, National Center for Emerging and Zoonotic Infectious Diseases, Centers for Disease Control and Prevention, Atlanta, GA; U.S. Public Health Service Commissioned Corps, Rockville, MD; Division of Healthcare Quality Promotion, National Center for Emerging and Zoonotic Infectious Diseases, Centers for Disease Control and Prevention, Atlanta, GA; Division of Healthcare Quality Promotion, National Center for Emerging and Zoonotic Infectious Diseases, Centers for Disease Control and Prevention, Atlanta, GA; U.S. Public Health Service Commissioned Corps, Rockville, MD

**Keywords:** COVID-19 vaccine, pregnancy, infant health, infant mortality

## Abstract

**Introduction:**

The COVID-19 Vaccine Pregnancy Registry (C19VPR) monitored pregnant women vaccinated for COVID-19 during December 2020 to June 2021. We report incidence of health outcomes among infants of C19VPR participants.

**Methods:**

Data about birthweight (e.g., low birthweight [LBW] <2,500 grams), small-for-gestational-age (SGA), death, and 10 prespecified conditions (i.e., cardiac arrest, cardiomyopathy, congenital hearing loss, heart failure, hypoxic-ischemic encephalopathy, intussusception, myocarditis/pericarditis, seizures, stroke, and supraventricular tachycardia) were collected through phone interviews from birth through 3 months postpartum; among a subsample, interviews extended through 15 months postpartum. Incidence of each prespecified condition was compared with prepandemic estimates.

**Results:**

Of 19,852 infants with 3-month follow-up data, 125 (0.6%) had at least one prespecified condition. Estimated C19VPR incidences for these conditions, LBW, SGA, and death, were within or below ranges reported in prepandemic studies.

**Conclusion:**

The incidence of LBW, SGA, death, or each of 10 prespecified infant conditions in C19VPR did not exceed prepandemic estimates.

## INTRODUCTION

During the pandemic, COVID-19 vaccination was recommended for U.S. residents aged 6 months and older, including pregnant women, to reduce the risk of severe COVID-19 illness (Centers for Disease Control and Prevention, 2024). Observational studies have demonstrated that COVID-19 vaccination in pregnancy confers protection from severe COVID-19 (e.g., leading to hospitalization or death) for both the mother and the newborn (Halasa et al., 2022; Jorgensen et al., 2023). In previous studies, vaccination has not been associated with adverse neonatal outcomes, such as higher rates of admission to neonatal care, congenital anomalies, preterm delivery, or neonatal mortality (Bernard et al., 2025; Jorgensen et al., 2023; Lipkind et al., 2022; Magnus et al., 2022; Sharma et al., 2025). However, information about broader infant health outcomes following COVID-19 vaccination during pregnancy is limited.

In a population-based cohort study enrolling 196,470 newborns in Norway and Sweden, COVID-19 vaccination in pregnancy did not increase risk of adverse neonatal outcomes, including mortality, bleeding or thrombotic conditions, disorders of the central nervous system, neonatal inflammation/infection, circulatory problems, respiratory distress, or gastrointestinal problems (Norman et al., 2024). In another study, lower neonatal morbidity was observed after vaccination during pregnancy compared to infants born to women who did not receive COVID-19 vaccines in pregnancy (Jorgensen et al., 2023). A study from the COVI-PREG registry conducted in Switzerland did not find increased risk for scoring low on a neurodevelopmental questionnaire at 12 months among infants born to mothers who received a COVID-19 vaccine during pregnancy compared to infants of mothers who did not (Favre et al., 2025).

The Centers for Disease Control and Prevention established the COVID-19 Vaccine Pregnancy Registry (C19VPR) to monitor health outcomes of mothers and infants following receipt of COVID-19 vaccines during or shortly before pregnancy (Madni et al., 2024). To better understand the incidence of a broad scope of infant health outcomes after COVID-19 vaccination during pregnancy, we assessed incidence of a range of health conditions among infants born to C19VPR participants.

## METHODS

### Study Design

V-safe is a vaccine safety monitoring system developed by the CDC to assess postvaccination symptoms of enrolled participants through internet-based surveys (Myers et al., 2023). The C19VPR is a convenience sample of women who were vaccinated from December 2020 to June 2021, voluntarily enrolled in V-safe during January to June 2021, and agreed to participate in C19VPR. All participants received at least one dose of the initial monovalent COVID-19 vaccine authorized for use in the United States (i.e., manufactured by Moderna, Pfizer-BioNTech, or Janssen/Johnson & Johnson) during pregnancy or up to 30 days before the last pregnancy-associated menstrual period. Structured phone interviews were planned for each trimester after enrollment, 4–8 weeks after the end of pregnancy, and when the infant was at least 3 months old. Interviews began in January 2021 and continued through August 2022. We asked participants about pre-existing and gestational health conditions, pregnancy outcome, delivery and postpartum complications, and infant health conditions through 3 months of age. Participants who consented to an extended follow-up were called at least 15 months after the end of pregnancy (November 2022 to September 2023) to complete a survey that included questions about infant health conditions diagnosed through 15 months of life. For simplicity, we use the term “infant” throughout, although health conditions could be diagnosed up to 15 months after birth for the follow-up. Details about C19VPR methodology have been published previously (Madni et al., 2024).

C19VPR was reviewed by the CDC and was conducted consistent with applicable federal law and CDC policy; the activity met requirements of public health surveillance as defined in 45 C.F.R. part 46.102(l)(2), 21 C.F.R. part 56; 42 U.S.C. §241(d); 5 U.S.C. §552a; 44 U.S.C. §3501 et seq.

### Outcomes: Infant Health Conditions

Data on infant birthweight and gestational age at birth were collected through phone interviews. Low birthweight (LBW) was defined as a birthweight <2,500 grams, regardless of gestational age at birth (Cutland et al., 2017). Birthweight and gestational age at birth were used to calculate each infant’s weight-for-gestational-age percentile. Infants were categorized as small-for-gestational-age (SGA) if they were below the tenth percentile in birthweight for their gestational age (Talge et al., 2014). Participants who reported that their infant was no longer living were asked age at infant death. Deaths prior to 28 days of life were categorized as neonatal deaths.

Each participant was asked whether their child had been diagnosed with any medical conditions by a healthcare provider. If the participant responded “yes,” the participant was asked for the name of the medical condition; whether any medications, treatment, procedures, or surgeries were received for this condition; and age at diagnosis. Participants were also asked whether their infant had been referred to any specialists and the reason for referral.

Data about infant health conditions were reviewed by two clinicians (LHZ and ES) who assigned a code based on the *International Statistical Classification of Diseases and Related Health Problems, Tenth Revision* Clinical Modification (ICD-10 CM) (World Health Organization, 2004) to infants with a reported health condition of interest In addition to LBW, SGA, and infant death (Cutland et al., 2017; Jorch et al., 2007; Schlaudecker et al., 2017), 10 prespecified health conditions (Table 1) were selected because they are key outcomes identified by the Brighton Collaboration (Brighton Collaboration, 2023) and Global Alignment of Immunization Safety Assessment (Bonhoeffer et al., 2016) or concerns expressed specifically about COVID-19 vaccination in children or adults (Yih et al., 2023). These conditions included 2 central nervous system disorders (nonfebrile seizures, hypoxic-ischemic encephalopathy), 5 cardiac conditions (supraventricular tachycardia, heart failure, cardiac arrest, myocarditis/pericarditis, cardiomyopathy), stroke, intussusception, and congenital hearing loss (Bonhoeffer et al., 2016; Carol Liu et al., 2020; Gollamudi et al., 2022; Sell et al., 2017). Prepandemic estimates derived with U.S. data were used as a comparator when available; if no U.S. estimates were available, incidences from other population-based studies were used.

Among 23,249 participants enrolled in C19VPR, 21,054 (90.6%) reported a live birth (Figure). Live birth was defined according to guidelines from the American Academy of Pediatrics and American College of Obstetricians and Gynecologists, which specify that live births can occur irrespective of the duration of pregnancy (Barfield, 2016). All infants from live births, including multiples (*n* = 21,378), were eligible for analysis for LBW, SGA, and infant death. To be eligible for analysis of infant health conditions diagnosed through 3 months, completion of an interview at least 3 months postpartum was required; 19,852 (92.9%) infants met this criterion. To be eligible for analysis of infant health conditions diagnosed through 15 months, the 15-month follow-up interview was required; 9,156 (42.8%) infants met this criterion.

Among participants who reported a prespecified infant health condition or infant death, we sought consent for medical record release from pediatric primary care providers and specialists. Two pediatric clinicians (LHZ and ES) abstracted medical records for each infant and classified each prespecified infant condition as confirmed, unconfirmed, or not present; discrepancies were resolved through discussion by clinicians. Infant conditions were confirmed if the medical record documented the diagnosis of the reported condition within the specified time frame (i.e., 3 or 15 months), unconfirmed if there was no evidence in the medical record of the diagnosis within the specified time frame, or ruled out if there was clear documentation that the infant did not have the condition (e.g., evaluated for a condition due to an initial concern but determined not to have the condition).

### Statistical Analysis

Descriptive statistics were estimated for demographics, pregnancy characteristics, pre-existing maternal health conditions, and infant characteristics. We calculated concordance of maternal report of infant conditions with medical record documentation. Using the proportion of cases with reported diagnoses confirmed through medical records, we calculated two estimates (“base” and “high”) of cases and rates per 1,000 live births. Base estimates were calculated by applying the total proportion of cases who were ruled out to the total number of maternal-reported cases and subtracting it from the total number of cases (maternal reports – (maternal reports × (ruled out/cases with records available)). For example, if a health condition had 10 maternal reports and one case was ruled out among 5 for which medical records were received, the base estimate would be 10 – (10 × (1/5)) = 8 cases. High estimates were calculated by assuming that all maternal-reported cases, except those that were ruled out through medical records, were true cases. In the previous example, the high estimate would be 9 cases. The incidence of each reported condition was calculated per 1,000 live births; 95% confidence intervals were estimated using exact methods (Agresti, 2018). All data were analyzed in SAS version 9.4 (SAS Institute, Cary, NC).

## RESULTS

In total, there were 21,054 C19VPR participants with 21,378 live births. Most participants were over 30 years of age (82.3%), non-Hispanic White (79.6%), and received an mRNA vaccine (59.1% Pfizer-BioNTech and 38.2% Moderna) (Table 2). COVID-19 vaccination was most common in the second trimester (40.5%), followed by the first trimester (26.1%), the third trimester (24.8%), and prepregnancy (8.7%). There were 383 (1.8%) multifetal pregnancies. Slightly less than half (48.4%) of infants were female, and 86.0% of infants were born at term (37–41 weeks’ gestation) (Table 3). Most infants were born vaginally (68.3%). A subset of 9,033 participants (42.9%) with 9,156 live-born infants participated in the follow-up through 15 months. Demographics of participants (Supplementary Table 1) and infants (Supplementary Table 2) were similar among those enrolled in C19VPR, those completing the 3-month postpartum interview, and those completing the 15-month follow-up.

The rates of LBW (72.6 per 1,000 live births, 95% CI: 69.2, 76.2), SGA (71.2 per 1,000 live births, 95% CI: 70.2, 77.4), and infant mortality (1.5 per 1,000 live births, 95% CI: 1.0–2.1) were lower than prepandemic rates based on natality data from 2023 National Vital Statistics System (Table 4) (Ely & Driscoll, 2024; National Center for Health Statistics, 2024; Schlaudecker et al., 2017). There were 32 reports of infant deaths occurring from birth through 3 months of life. Among the 32 infants who died, 20 (62.5%) were preterm, including four (12.5%) born alive prior to 20 weeks’ gestation. Primary reasons for infant death included prematurity (*n* = 9, 28.1%), birth defect or genetic anomaly (*n* =8, 25%), sepsis (*n* = 3, 9.4%), hypoxic ischemic encephalopathy (*n* = 3, 9.4%), sudden unexpected infant death (*n* = 2, 6.3%), accident (*n* = 1, 3.1%), disseminated intravascular coagulation (*n* = 1, 3.1%) and unknown (*n* = 5, 15.6%). Among the five infants with an unknown reason for death, one was born at 23 weeks’ gestation, one at 29 weeks’ gestation, and three were full-term. Twenty-four deaths (75.0%) were neonatal deaths. One additional infant death was reported at the 15-month follow-up and was attributed to a birth defect. Medical records were received for 25 (78.1%) of the 32 reported infant deaths; all 25 were confirmed deaths.

Among 19,852 (92.9%) infants whose mothers completed the 3-month postpartum interview, 125 (0.6%) infants were reported to have been diagnosed with at least one of the 10 prespecified infant health conditions prior to 3 months; 13 infants had more than one health condition (0.07%) (Table 5). Among the 13 infants with more than one health condition, 11 (84.6%) were reported to have had a seizure along with another medical condition. Medical records were received for 87 (69.6%) of the 125 infants with maternal report of a health condition prior to 3 months, including 10 infants with more than one condition, for 97 reports of prespecified health conditions. Of the 97 reports of prespecified health conditions, 59 (60.8%) were confirmed through medical records, 25 (25.8%) were unable to be confirmed, and 13 (13.4%) had medical record documentation that the infant did not have the reported condition (i.e., ruled out).

Among infants whose health conditions were not ruled out during review of medical records (i.e., either were confirmed or were unable to be confirmed) during review of medical records, the most reported health conditions were seizures (1.8–1.9 per 1,000 live births), hearing loss (1.5 –1.7 per 1,000 live births), hypoxic-ischemic encephalopathy (1.0 per 1,000 live births), and supraventricular tachycardia (0.7 per 1,000 live births). There were 10 cases of heart failure (0.5 per 1,000 live births), six cases of stroke (0.3 per 1,000 live births), four cases of cardiomyopathy (0.2 per 1,000 live births), and two cases of cardiac arrest (0.1 per 1,000 live births). There were no reports of intussusception or myocarditis/pericarditis among infants aged 0–3 months. The C19VPR incidence of 8 infant medical conditions with an available comparator were within or below the prepandemic estimate (Centers for Disease Control and Prevention, 2023; Chu et al., 2015; Cornet et al., 2023; deVeber et al., 2017; Dunbar et al., 2020; Lipshultz et al., 2003; Ludomirsky & Garson, 1990; Padiyar et al., 2020; Pisani et al., 2018; Tate et al., 2016; Vasudevan & Levene, 2013). There were no available published comparators for cardiac arrest or heart failure.

Of the 9,156 infants with available data from the 15-month follow-up, 110 (1.2%) were reported to have one of the 10 prespecified infant health condition diagnosed between 4 and 15 months of life; four infants had two health conditions (Table 6). Medical records were received for 77 infants (70.0%) with 77 reports of health conditions. Of the 77 reports of health conditions, 10 (13.0%) were confirmed through medical records, 19 (24.6%) were unable to be confirmed, and 48 (62.3%) had medical record documentation that the infant did not have the reported condition; 47 of the 48 reports that were ruled out were reports of hearing loss.

Among infants whose health conditions were not ruled out during medical record review, the most reported infant medical conditions reported during the 15-month follow-up were seizures (2.8 per 1,000 live births), hearing loss (1.6–3.2 per 1,000 live births), intussusception (0.4 per 1,000 live births), and supraventricular tachycardia (0.4 per 1,000 live births). There was one additional unconfirmed report of stroke. There were no new reports of cardiac arrest, cardiomyopathy, heart failure, myocarditis/pericarditis, or hypoxic-ischemic encephalopathy in the 4–15-month follow-up. Incidence among C19VPR infants was within or below the prepandemic incidence for supraventricular tachycardia, intussusception, and cardiomyopathy (Centers for Disease Control and Prevention, 2023; Chu et al., 2015; Lipshultz et al., 2003; Ludomirsky & Garson, 1990; Tate et al., 2016). There were no available comparator rates for stroke or seizures occurring in infancy outside the neonatal period.

## DISCUSSION

The C19VPR was established soon after authorization of COVID-19 vaccines in the United States as part of a comprehensive approach to conduct postmarketing safety surveillance on COVID-19 vaccines during pregnancy (Centers for Disease Control and Prevention, 2025a; Madni et al., 2024). In our cohort of 21,378 infants, we found no evidence of increased incidence for LBW, SGA, or infant death compared to data from the 2023 National Vital Statistics System. Among 19,852 infants with data on infant health conditions diagnosed through 3 months of age, we found no evidence of increased incidence of each of 10 prespecified infant health conditions. These findings may be helpful to public health professionals, nurses, and pediatric and family nurse practitioners and inform vaccine discussions between clinicians and pregnant women and their families.

Our data are consistent with previous studies that have failed to find an association between COVID-19 vaccination during pregnancy and neonatal mortality (Jorgensen et al., 2023; Lindsay et al., 2023; Norman et al., 2024). Neonatal mortality in C19VPR was 1.2 infant deaths per 1,000 live births, lower than the background rate (3.7 deaths per 1,000 infants in 2023) (Ely & Driscoll, 2024). Most causes of death were associated with extreme prematurity or a fatal birth defect or genetic anomaly, findings consistent with previous research on leading causes of infant deaths (Kochanek et al., 2024). No association between COVID-19 vaccination and preterm birth or birth defects was found in other studies (Ding et al., 2023; Kharbanda et al., 2024; Lindsay et al., 2023; Lipkind et al., 2022; Magnus et al., 2024; Raffetti et al., 2024; Ruderman et al., 2022). Our observation could reflect some healthy vaccine effects and perhaps other forms of residual confounding.

We observed no increase in LBW or SGA among infants of mothers vaccinated against COVID-19 during pregnancy. In a Vaccine Safety Datalink study, COVID-19 vaccination during pregnancy was not associated with risk of SGA among 40,627 live births with recorded birthweight (aHR: 0.95; 95% CI: 0.87–1.03) (Lipkind et al., 2022). Findings were consistent when stratified by type of COVID-19 vaccination and by trimester of vaccination (Lipkind et al., 2022). Similar findings were observed in a population-based study conducted in Ontario, Canada, among >85,000 infants (Fell et al., 2022) and in a Scandinavian birth registry-based study including 157,521 deliveries (Magnus et al., 2022).

Cardiac-related diagnoses, specifically myocarditis and pericarditis, have been associated with mRNA COVID-19 vaccination in children and young adults, particularly among males (Oster et al., 2022). We identified no cases of infant myocarditis or pericarditis in our study. In a previous study including 94,303 participants vaccinated during pregnancy in Sweden and Norway, no cases of neonatal myocarditis were identified (Norman et al., 2024). We evaluated several other cardiac conditions, including cardiac arrest, cardiomyopathy, heart failure, and supraventricular tachycardia. Estimated C19VPR incidence rates for cardiomyopathy and supraventricular tachycardia were below published prepandemic estimates (Chu et al., 2015; Lipshultz et al., 2003; Ludomirsky & Garson, 1990). However, published estimates for supraventricular tachycardia were among neonates discharged from neonatal intensive care units, which might not be directly comparable to our cohort (Chu et al., 2015). It is also possible that incidence of cardiomyopathy among infants has changed since estimates were published in 2003 (Lipshultz et al., 2003); more recent population-based data on incidence of infant cardiomyopathy could not be identified. We were unable to identify comparator estimates in the literature for cardiac arrest and heart failure. One previous study including all infants in Sweden and Norway born from June 2021 to January 2023 found no associations between circulatory conditions, including heart failure or cardiac ischemia, among infants born to COVID-19 vaccinated mothers (Norman et al., 2024).

Thrombosis with thrombocytopenia has been reported as a rare complication of vaccination with adenovirus-vector COVID-19 vaccines in adults (Pottegård et al., 2021; See et al., 2021). In our study, thrombotic adverse events were rare. The rate of stroke was 0.3 per 1,000 live births, below prepandemic Canadian estimates of 0.8–1.2 neonatal strokes per 1,000 births (deVeber et al., 2017; Dunbar et al., 2020). These findings add to evidence from a population-based study from Sweden and Norway enrolling 196,470 newborns that found no risk of cerebral infarction among infants whose mothers received COVID-19 vaccination, regardless of the trimester in which the vaccination was received (Norman et al., 2024). Norman et al. observed that vaccination in the second trimester was associated with reduced odds of hypoxic-ischemic encephalopathy. Neonatal convulsions were not associated with maternal vaccination in any trimester (Norman et al., 2024). Incidence of hypoxic-ischemic encephalopathy in C19VPR was similar to the published rate among neonates born >35 weeks’ gestation during 2012–2019 in North California Kaiser Permanente Hospitals (Cornet et al., 2023). Incidence of febrile seizures in C19VPR was similar to published rates from studies conducted in the United States, Italy, and Canada (Cornet et al., 2023; Padiyar et al., 2020; Pisani et al., 2018; Vasudevan & Levene, 2013).

Intussusception has been reported in case reports of infants infected with SARS-CoV-2, but no study has examined potential associations between maternal or infant COVID-19 immunization and intussusception (Scottoni et al., 2022). No cases of intussusception were reported among infants followed for 3 months in C19VPR. Four cases of intussusception were reported during the 15-month follow-up (0.4 per 1,000 live births). This estimate is substantially lower than the 2007–2013 rate estimate of 3.4–4.0 per 1,000 live births from the U.S among infants. Healthcare Cost and Utilization Project State Inpatient Database (Tate et al., 2016). It is possible that we observed a lower rate due to reporting bias or because the rate of acute gastroenteritis was lower during the pandemic compared to prepandemic periods because of increased handwashing or social distancing policies (Centers for Disease Control and Prevention, 2025b; Parrôn et al., 2023; Saidian et al., 2025). Acute gastroenteritis among children aged 3 months to 3 years is a well-established risk factor for intussusception (Burnett et al., 2020).

Early in the pandemic, there were several case reports of sensorineural hearing loss following SARS-CoV-2 infection and following mRNA and adenovirus vector-based COVID-19 vaccination, though no population-level associations have been identified (Nieminen et al., 2023). Among our C19VPR cohort of infants, incidence of congenital hearing loss was similar (1.5–1.7 per 1,000 live births) to the incidence of hearing loss identified by the U.S. Early Hearing Detection and Intervention Program (1.7 per 1,000 live births) (Centers for Disease Control and Prevention, 2023). Reports of incident hearing loss during the 4–15-month follow-up period were not uncommon. Among 47 of 58 (81.0%) infants whose medical records were reviewed, hearing loss was temporary and most often related to recurrent otitis media. Three (5.2%) were confirmed congenital hearing loss, and eight (13.8%) were unable to be confirmed.

This cohort study of infants has several strengths, including a sample size of >20,000 infants whose mothers received the COVID-19 vaccine during pregnancy. We explored potential associations between maternal vaccination and a broad range of infant medical conditions. This study has several limitations. Although we had a large sample size, the study had limited ability to reliably observe rare outcomes with expected prevalences below 1.5 per 10,000 infants (Gupta et al., 2016). Because C19VPR included only exposed (vaccinated) women but no unvaccinated women, rates for health conditions of interest can only compared with previously published rates in the general population. However, interpretation of these comparisons is limited because many of the published estimates are more than a decade old and newer population-based estimates are unavailable. Criteria used to diagnose conditions and demographic and clinical characteristics may differ between the C19VPR cohort and the populations from which background rates were derived. The C19VPR relied on maternal-reported data. C19VPR is also relatively homogenous with respect to race, ethnicity, and maternal age and is not a nationally representative sample.

## CONCLUSION

In a cohort of nearly 20,000 infants whose mothers were vaccinated in pregnancy, we did not identify increases in LBW, SGA, infant mortality, or 10 selected infant health conditions: cardiac arrest, cardiomyopathy, congenital hearing loss, heart failure, hypoxic-ischemic encephalopathy, intussusception, myocarditis/pericarditis, seizure, stroke, or supraventricular tachycardia. These findings can help inform pediatric healthcare providers and nurses in their discussions with parents who may be concerned about health conditions of their children following maternal COVID-19 vaccination.

## Supplementary Material

Supplementary Table 2

Supplementary Table 1

Supplementary material associated with this article can be found in the online version at https://doi.org/10.1016/j.pedhc.2026.06.017.

## Figures and Tables

**FIGURE.**
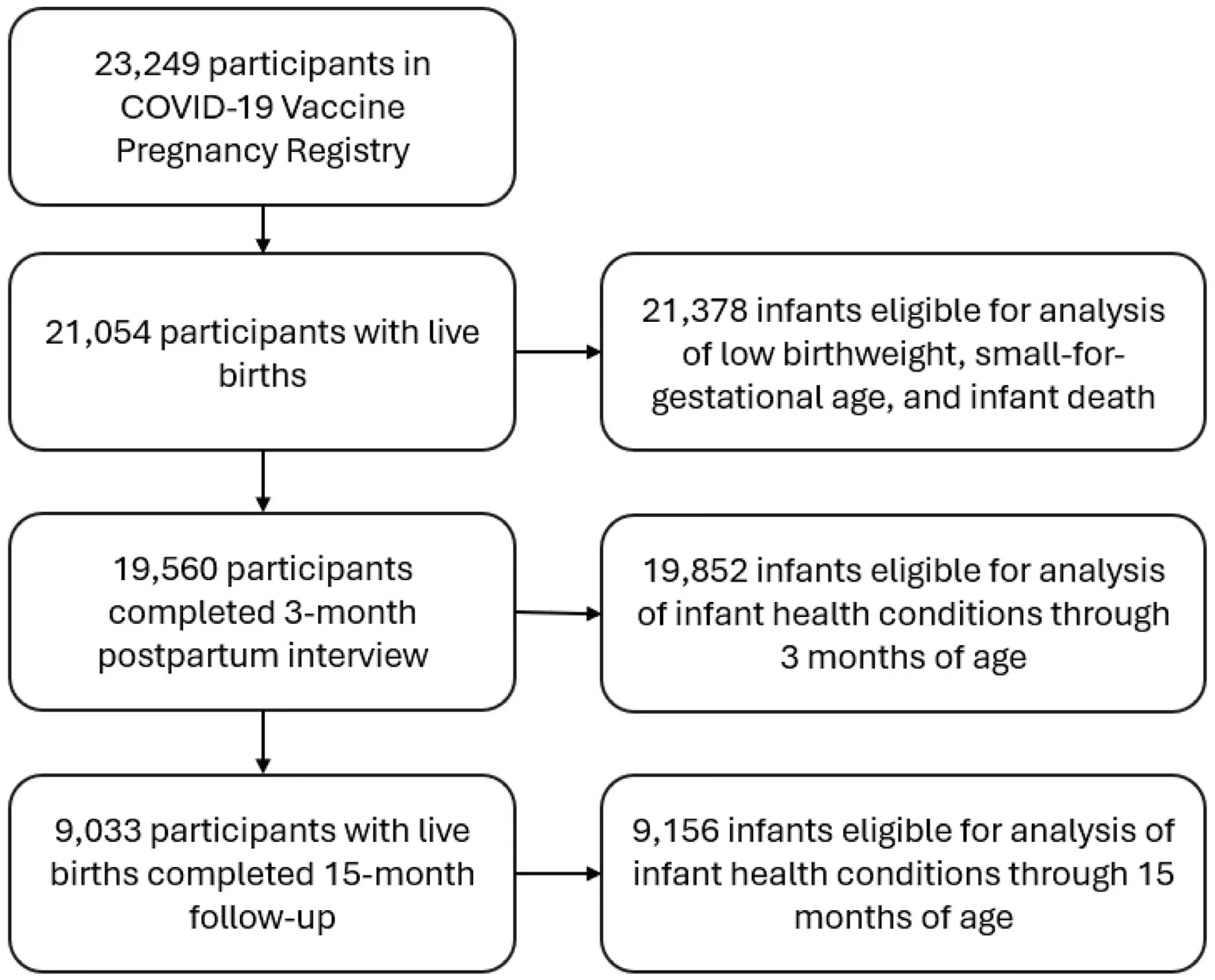
Consort diagram of COVID-19 Vaccine Pregnancy Registry.

**TABLE 1.** List of prespecified infant health outcomes and conditions^a^

Infant outcomes
Small-for-gestational age Low birthweight Infant mortality
Infant health conditions
Neurological disorders Hypoxic ischemic encephalopathy Nonfebrile seizures Cardiac disorders Cardiac arrest Cardiac arrhythmia (i.e., supraventricular tachycardia) Cardiomyopathy Heart failure Myocarditis/pericarditis Stroke Congenital hearing loss Intussusception

a Key outcomes identified by the Brighton Collaboration, Global Alignment of Immunization Safety Assessment, or concerns expressed specifically about COVID-19 vaccination in children or adults.

**TABLE 2.** Demographics of participants with live births enrolled in the COVID-19 Vaccine Pregnancy Registry, January 2021 to August 2022

	C19VPR (*n* = 21,054)	
	*n*	%
**Age at vaccination (years)**		
18–24	344	1.6
25–29	3,375	16.0
30–34	10,253	48.7
35–39	6,142	29.2
40+	940	4.5
**Race and ethnicity**		
Non-Hispanic Black	462	2.2
Non-Hispanic White	16,753	79.6
Hispanic	1,950	9.3
Other	1,851	8.9
Missing	38	0.2
**Prepregnancy body mass index (kg/m^2^)**		
<18.5	343	1.6
18.5–24.9	10,786	51.2
25.0–29.9	5,348	25.4
≥30.0	4,072	19.3
Missing	505	2.4
**Pre-existing conditions**		
Diabetes	206	1.0
Hypertension	886	4.2
**Manufacturer of COVID-19 vaccine**		
Pfizer-BioNTech	12,436	59.1
Moderna	8,035	38.2
Janssen	583	2.8
**Trimester of registry-eligible dose**		
Prepregnancy	1,826	8.7
First trimester	5,491	26.1
Second trimester	8,525	40.5
Third trimester	5,212	24.8
**Multiple gestation pregnancy**		
Yes	383	1.8
**Parity**		
0	8,850	42.0
1	7,925	37.6
2–8	3,653	17.4
Missing	626	3.0
**Healthcare worker**		
No	11,775	55.9
Yes	8,965	42.6
Missing	314	1.5
Urban residence		
No	1,324	6.3
Yes	19,697	93.6
Missing	33	0.2

**TABLE 3.** Characteristics of infants of participants enrolled in COVID-19 Vaccine Pregnancy Registry, January 2021 to August 2022

	C19VPR (*n* = 21,378)	
	*n*	%
**Infant sex**		
Male	10,930	51.1
Female	10,341	48.4
Missing	107	0.5
**Gestational age at birth (weeks)**		
<28	56	0.3
28 to <32	135	0.6
32 to <34	197	0.9
34 to <37	1,292	6.0
37 to <41	18,381	86.0
41+	1,312	6.1
Missing	5	0.0
**Birthweight (grams)**		
<1,500	148	0.7
1,500–2,499	1,019	4.8
2,500–3,999	18,049	84.4
4,000+	1,776	8.3
Missing	386	1.8
**Delivery method**		
Vaginal	14,609	68.3
Cesarean section	6,687	31.3
Missing	82	0.4

**TABLE 4.** Reported infant health outcomes among live-born infants of COVID-19 Vaccine Pregnancy Registry participants (*n* = 21,378), January 2021 to August, 2022

	C19VPR maternal report		Background Rate
	n	Rate per 1,000 live births	Rate per 1,000 live births
Low birthweight (<2,500 g)	1,553	72.6 (69.2, 76.2)	86^a^
Small-for-gestational-age	1,524	71.2 (70.2, 77.4)	100^b^
Infant mortality^c^	32	1.5 (1.0, 2.1)	5.6^d^
Neonatal mortality	24	1.1 (0.7, 1.7)	3.7^d^

a National Center for Health Statistics (2024); final natality data for 2023.

b Talge et al. (2014); SGA is defined as <10th percentile for weight-and-gestational age at birth. By definition, approximately 100 infants would be SGA.

c C19VPR infant mortality rate is for 0–3 months; the background rate is for 0–12 months. C19VPR had an additional 1 reported death in the 4–15-month follow-up for a total of 33 deaths. We could not calculate an infant mortality rate because there were a different number of participants in the 4–15-month follow-up data, so the denominator is different. Therefore, these rates are not directly comparable.

d Ely & Driscoll (2024); data from 2023 using U.S. linked birth/infant death files from National Center for Health Statistics; this rate is for 0–12 months, whereas C19VPR maternal report is 0–3 months.

**TABLE 5.** Reported and medical record confirmation of infant health conditions diagnosed 0–3 months among live-born infants of COVID-19 Vaccine Pregnancy Registry participants who completed a postpartum interview after 3 months (*n* = 19,852), January 2021 to August 2022

	Maternal report		Medical record determination			Estimated true cases^b^		Estimated rate per 1,000 live births^b^		Prepandemic rate per 1,000 live births
	*n*	# records received	Confirmed *n* (%)	Unconfirmed *n* (%)	Ruled out *n* (%)	Base (*n*)	High (*n*)	Base	High	
Infant condition^a^	125									
Cardiac arrest	2	2	1 (50.0)	1 (50.0)	0	2	2	0.1	0.1	–
Cardiomyopathy	6	6	1 (16.7)	3 (50.0)	2 (33.4)	4	4	0.2	0.2	0.8^c^
Hearing loss	41	27	15 (55.6)	5 (18.5)	7 (25.9)	30.4	34	1.4	1.7	1.8^d^
Heart failure	10	7	2 (28.6)	5 (71.4)	0	10	10	0.5	0.5	–
Hypoxic-Ischemic Encephalopathy	20	12	12 (100.0)	0	0	20	20	1.0	1.0	1.7^e^
Intussusception	0	–	–	–	–	0	0	0	0	1.7–2.3^f^
Myocarditis/Pericarditis	0	–	–	–	–	0	0	0	0	0
Seizures (nonfebrile)	40	26	14 (54.0)	9 (34.6)	3 (11.5)	35.4	37	1.8	1.9	0.4–3^g,h,i^
Stroke	7	5	3 (60.0)	1 (20.0)	1 (20.0)	5.6	6	0.3	0.3	0.8–1.2^j,l^
Supraventricular tachycardia	14	12	11 (91.7)	1 (8.3)	0	14	14	0.7	0.7	3^l^

a 12 infants had two conditions, 1 infant had 3 conditions; of the 13 infants with more than one condition, medical records were received for 10.

b Estimated true case range: Base estimate, the proportion of ruled out was applied to the total number of reported cases; high, only the number of ruled out cases excluded; any unconfirmed cases with medical records and all cases without medical records received considered true cases.

c Lipshultz et al. (2003); pediatric cardiomyopathy registry in New England and Central Southwest Region of the United States, numerators included patients <1 year who received a diagnosis of cardiomyopathy between 1996 and 1999, denominator included 1990 U.S. Census.

d Centers for Disease Control and Prevention (2022); prevalence of hearing loss among U.S. infants screened for hearing loss in 2022.

e Cornet et al. (2023); includes incidence of hypoxic ischemic encephalopathy among infants born ≥35 weeks’ gestation at Northern California Kaiser Permanente hospitals from 2012 to 2019.

f Tate et al. (2016); incidence of intussusception among infants 6–14 weeks old, U.S. Healthcare Cost and Utilization Project’s State Inpatient Database from 2007 to 2013.

g Padiyar et al., 2020; includes only neonatal seizures among admitted infants; data from the Kids’ Inpatient Database for 2006, 2009, 2012.

h Vasudevan and Levene (2013); includes only neonatal seizures, estimation based on six population-based studies in the United States and Newfoundland, Canada.

i Pisani et al. (2018); includes neonatal polygraphic video electroencephalogram-confirmed seizures at hospital in Parma, Italy, during January 2002 to December 2014 among both preterm and term infants; denominator included all live births in Parma province.

j deVeber et al. (2017); includes arterial ischemic strokes in neonates diagnosed at any of the 16 Canadian acute care pediatric hospitals from January 1992 to December 2001; national population estimates for live births were obtained through Statistics Canada.

k Dunbar et al. (2020); includes any neuroimaging-confirmed perinatal stroke among neonates born >36 weeks’ gestation from 2008 to 2017 among all live births and stillbirths in Southern Alberta, Canada.

l Chu et al. (2015); includes prevalence of SVT among infants discharged from Pediatrix Medical Group neonatal intensive care units between 1998 and 2012 with a diagnosis of SVT.

**TABLE 6.** Reported and medical record confirmation of infant health conditions diagnosed at 4–15 months of life among infants of COVID-19 Vaccine Pregnancy Registry participants who participated in the 15-month follow-up of data collection (*n* = 9,156), November 2022 to September, 2023

	Maternal report		Medical record determination			Estimated true cases^b^		Estimated rate per 1,000 live births		Prepandemic rate per 1,000 live births
	n	# records received	Confirmed *n* (%)	Unconfirmed *n* (%)	Ruled out *n* (%)	Base (*n*)	High (*n*)	Base	High	
Infant condition^a^	110									
Cardiac arrest	0	0	0	0	0	0	0	–	–	–
Cardiomyopathy	0	0	0	0	0	0	0	–	–	0.8^c^
Hearing loss	78	58	3 (5.2)	8 (13.8)	47 (81.0)	14.4	29	1.6	3.2	1.7^d^
Heart failure	0	0	0	0	0	0	0	–	–	–
Hypoxic-Ischemic Encephalopathy	0	0	0	0	0	0	0	–	–	–
Intussusception	4	1	0	1 (100.0)	0	4	4	0.4	0.4	3.4–4.0^e^
Myocarditis/Pericarditis	0	0	0	0	0	0	0	–	–	–
Seizures (nonfebrile)	26	13	5 (38.5)	8 (61.5)	0	26	26	2.8	2.8	–
Stroke	1	1	0	1 (100.0)	0	1	1	0.1	0.1	–
Supraventricular tachycardia	5	4	2 (50.0)	1 (25.0)	1 (25.0)	3.8	4	0.4	0.4	3^f^

a 4 infants had 2 conditions reported; medical records were not received for these 4 infants.

b Estimated true case range: Base estimate, the proportion of ruled out was applied to the total number of reported cases; high, only the number of ruled out cases excluded; any unconfirmed cases with medical records and all cases without medical records received considered true cases.

c Lipshultz et al. (2003); pediatric cardiomyopathy registry in New England and Central Southwest Region of the United States; numerators included patients <1 year who received a diagnosis of cardiomyopathy between 1996 and 1999, denominator included 1990 U.S. Census.

d Centers for Disease Control and Prevention (2022); prevalence of hearing loss among U.S. infants screened for hearing loss in 2022.

e Tate et al. (2016); incidence of intussusception among infants <12 months old, U.S. Healthcare Cost and Utilization Project’s State Inpatient Database from 2007 to 2013.

f Chu et al. (2015); includes prevalence of SVT among infants discharged from Pediatrix Medical Group neonatal intensive care units between 1998 and 2012 with a diagnosis of SVT.

## Data Availability

Assurance of confidentiality restrictions apply to these data. To apply for the study data, please contact the corresponding author.
