# Supplementary material for: Neonatal and Infant Health Outcomes Following Maternal COVID-19 Vaccination: CDC’s COVID-19 Vaccine Pregnancy Registry
Source: J Pediatr Health Care. Author manuscript; Available in PMC 2026 Oct 5. (PMC13636450; doi:10.1016/j.pedhc.2026.06.017)
Supplement: Supplementary file 1 [file NIHMS2210744-supplement-Supplementary_Table_1.docx]

**Supplemental materials**

**Supplemental Table 1.** Demographics of participants with live births enrolled in the COVID-19 Vaccine Pregnancy Registry, those who completed a 3-month postpartum survey, January 2021- August 2022, and those who completed a 15 month follow-up survey, November 2022 - September 2023

|  | **Participants with live births (n=21,054)** | | **Participants completing a 3-month postpartum interview (n=19,560)** | | **Participants completing a 15-month postpartum interview (n=9,033)** | |
| --- | --- | --- | --- | --- | --- | --- |
|  | N | % | N | % | N | % |
| **Age at vaccination (years)** |  |  |  |  |  |  |
| 18-24 | 344 | 1.6 | 306 | 1.6 | 150 | 1.7 |
| 25-29 | 3,375 | 16.0 | 3,033 | 15.5 | 1,404 | 15.5 |
| 30-34 | 10,253 | 48.7 | 9,554 | 48.8 | 4,346 | 48.1 |
| 35-39 | 6,142 | 29.2 | 5,772 | 29.5 | 2,692 | 29.8 |
| 40+ | 940 | 4.5 | 895 | 4.6 | 441 | 4.9 |
| **Race and ethnicity** |  |  |  |  |  |  |
| Non-Hispanic Black | 462 | 2.2 | 380 | 1.9 | 212 | 2.4 |
| Non-Hispanic White | 16,753 | 79.6 | 15,671 | 80.1 | 7,193 | 79.6 |
| Hispanic | 1,950 | 9.3 | 1,772 | 9.1 | 838 | 9.3 |
| Other | 1,851 | 8.9 | 1,703 | 8.7 | 778 | 8.6 |
| Missing | 38 | 0.2 | 34 | 0.2 | 12 | 0.1 |
| **Pre-pregnancy body mass index (kg/m^2^)** |  |  |  |  |  |  |
| <18.5 | 343 | 1.6 | 328 | 1.7 | 138 | 1.5 |
| 18.5-24.9 | 10,786 | 51.2 | 10,125 | 51.8 | 4,599 | 50.9 |
| 25.0-29.9 | 5,348 | 25.4 | 4,980 | 25.5 | 2,330 | 25.8 |
| ≥30.0 | 4,072 | 19.3 | 3,770 | 19.3 | 1,846 | 20.4 |
| Missing | 505 | 2.4 | 357 | 1.8 | 120 | 1.3 |
| **Pre-existing conditions** |  |  |  |  |  |  |
| Diabetes | 206 | 1.0 | 191 | 1.0 | 90 | 1.0 |
| Hypertension | 886 | 4.2 | 818 | 4.2 | 400 | 4.4 |
| **Manufacturer of COVID-19 vaccine** |  |  |  |  |  |  |
| Pfizer-BioNTech | 12,436 | 59.1 | 11,659 | 59.6 | 5,363 | 59.4 |
| Moderna | 8,035 | 38.2 | 7,410 | 37.9 | 3,421 | 37.9 |
| Janssen | 583 | 2.8 | 491 | 2.5 | 249 | 2.8 |
| **Trimester of registry-eligible dose** |  |  |  |  |  |  |
| Pre-pregnancy | 1,826 | 8.7 | 1,397 | 7.2 | 799 | 8.8 |
| First trimester | 5,491 | 26.1 | 4,996 | 25.5 | 2,419 | 26.8 |
| Second trimester | 8,525 | 40.5 | 8,205 | 42.0 | 3,669 | 40.6 |
| Third trimester | 5,212 | 24.8 | 4,962 | 25.4 | 2,146 | 23.8 |
| **Multiple gestation pregnancy** |  |  |  |  |  |  |
| Yes | 383 | 1.8 | 346 | 1.8 | 143 | 1.6 |
| **Parity** |  |  |  |  |  |  |
| 0 | 8,850 | 42.0 | 8,267 | 42.3 | 3,916 | 43.4 |
| 1 | 7,925 | 37.6 | 7,462 | 38.2 | 3,388 | 37.5 |
| 2-8 | 3,653 | 17.4 | 3,375 | 17.3 | 1,525 | 16.9 |
| Missing | 626 | 3.0 | 456 | 2.3 | 204 | 2.3 |
| **Healthcare worker** |  |  |  |  |  |  |
| No | 11,775 | 55.9 | 11,233 | 57.4 | 5,318 | 58.9 |
| Yes | 8,965 | 42.6 | 8,160 | 41.7 | 3,685 | 40.8 |
| Missing | 314 | 1.5 | 167 | 0.9 | 30 | 0.3 |
| **Urban residence** |  |  |  |  |  |  |
| No | 1,324 | 6.3 | 1,201 | 6.1 | 579 | 6.4 |
| Yes | 19,697 | 93.6 | 18,330 | 93.7 | 8,444 | 93.5 |
| Missing | 33 | 0.2 | 29 | 0.2 | 10 | 0.1 |
