# Supplementary material for: Neonatal and Infant Health Outcomes Following Maternal COVID-19 Vaccination: CDC’s COVID-19 Vaccine Pregnancy Registry
Source: J Pediatr Health Care. Author manuscript; Available in PMC 2026 Oct 5. (PMC13636450; doi:10.1016/j.pedhc.2026.06.017)
Supplement: Supplementary file 2 [file NIHMS2210744-supplement-Supplementary_Table_2.docx]

**Supplemental Table 2.** Characteristics of infants of participants enrolled in COVID-19 Vaccine Pregnancy Registry and completing a 3-month postpartum survey, January 2021- August 2022, and 15-month follow-up survey, November 2022 - September 2023

|  | **Infants of participants with live births (n=21,378)** | | **Infants of participants completing a 3-month follow-up survey (n=19,852)** | | **Infants of participants completing a 15-month follow-up survey (n=9,156)** | |
| --- | --- | --- | --- | --- | --- | --- |
|  | n | % | n | % | n | % |
| **Infant Sex** |  |  |  |  |  |  |
| Male | 10,930 | 51.1 | 10,140 | 51.1 | 4,684 | 51.2 |
| Female | 10,341 | 48.4 | 9,655 | 48.6 | 3,437 | 48.5 |
| Missing | 107 | 0.5 | 57 | 0.3 | 35 | 0.4 |
| **Gestational age at birth (weeks)** |  |  |  |  |  |  |
| <28 | 56 | 0.3 | 49 | 0.3 | 28 | 0.3 |
| 28-<32 | 135 | 0.6 | 126 | 0.6 | 54 | 0.6 |
| 32-<34 | 197 | 0.9 | 184 | 0.9 | 81 | 0.9 |
| 34-<37 | 1,292 | 6.0 | 1,188 | 6.0 | 555 | 6.1 |
| 37-<41 | 18,381 | 86.0 | 17.083 | 86.1 | 7,872 | 86.0 |
| 41+ | 1,312 | 6.1 | 1,220 | 6.2 | 564 | 6.2 |
| Missing | 5 | 0.0 | 3 | 0.0 | 2 | 0.0 |
| **Birthweight (grams)** |  |  |  |  |  |  |
| <1,500 | 148 | 0.7 | 138 | 0.7 | 66 | 0.7 |
| 1,500-2,499 | 1,019 | 4.8 | 942 | 4.8 | 446 | 4.9 |
| 2,500g-3,999 | 18,049 | 84.4 | 16,816 | 84.7 | 7,707 | 84.2 |
| 4,000+ | 1,776 | 8.3 | 1,655 | 8.3 | 785 | 8.6 |
| Missing | 386 | 1.8 | 301 | 1.5 | 152 | 1.7 |
| **Delivery Method** |  |  |  |  |  |  |
| Vaginal | 14,609 | 68.3 | 13,618 | 68.6 | 6,296 | 68.8 |
| Cesarean section | 6,687 | 31.3 | 6,189 | 31.2 | 2,837 | 31.0 |
| Missing | 82 | 0.4 | 45 | 0.2 | 22 | 0.2 |
